# Association of smoking with amyotrophic lateral sclerosis risk and survival in men and women: a prospective study

**DOI:** 10.1186/1471-2377-10-6

**Published:** 2010-01-14

**Authors:** Alvaro Alonso, Giancarlo Logroscino, Susan S Jick, Miguel A Hernán

**Affiliations:** 1 Division of Epidemiology and Community Health, School of Public Health, University of Minnesota, Minneapolis, MN, USA; 2 Department of Preventive Medicine and Public Health, School of Medicine, University of Navarra, Pamplona, Spain; 3 Department of Neurological Sciences, University of Bari, Bari, Italy; 4 Boston Collaborative Drug Surveillance Program, Boston University, Lexington, MA, USA; 5 Department of Epidemiology, Harvard School of Public Health, Boston, MA, USA; 6 Harvard-MIT Division of Health Sciences and Technology, Boston, MA, USA

## Abstract

**Background:**

Previous epidemiologic studies have examined the association of smoking with amyotrophic lateral sclerosis (ALS) incidence, but their results have been inconsistent. Moreover, limited information exists on the association between smoking and survival in ALS patients. We evaluated the association of smoking with ALS incidence and survival in a population-based cohort.

**Methods:**

We conducted a case-control study nested in the General Practice Research Database, a computerized clinical database in the United Kingdom. Cases were 1143 individuals with a diagnosis of ALS; 11,371 matched controls were selected among GPRD participants free of ALS. Predictors of survival were determined in the ALS cases. Smoking information was obtained from the computer database.

**Results:**

Smoking was not associated with the risk of ALS in this population. The rate ratio (RR) of ALS comparing ever versus never smokers was 1.04, 95% confidence interval (CI) 0.80-1.34. In analysis stratified by gender, however, ever smoking was associated with ALS in women (RR 1.53, 95% CI 1.04-2.23) but not in men (RR 0.75, 95% CI 0.53-1.06). Mortality was 71% after 2.1 average years of follow-up. Old age and female sex were associated with lower survival. Smoking was a predictor of mortality only in women. Comparing ever versus never smokers, RR (95% CI) of death was 1.31 (1.04-1.65) in women, and 0.90 (0.72-1.11) in men.

**Conclusion:**

In this large population-based study, smoking was associated with ALS risk and worse survival in women but not in men.

## Background

Amyotrophic lateral sclerosis (ALS) is a neurodegenerative disease characterized by the progressive loss of upper and lower motor neurons. Although its etiology remains unknown, certain environmental (non-genetic) factors have been postulated as potential causative factors[1]. Cigarette smoking, in particular, has attracted interest as a risk factor for ALS. Smoking could increase the risk of ALS through several mechanisms, including inflammation, oxidative stress, and neurotoxicity caused by heavy metals and other chemical compounds present in cigarette smoke[2-5].

A number of epidemiologic studies have assessed the association of cigarette smoking with ALS incidence, but their results have been inconsistent[6]. More recently, a systematic review and meta-analysis has suggested that sex could modify the association between smoking and ALS risk, with smoking linked to higher risk of ALS in women but not in men[7]. Similarly, the only three studies that estimated the association of smoking with the survival of ALS patients were inconclusive[8-10].

Here we present estimates of the association of smoking with ALS risk, and with survival in ALS patients, in the General Practice Research Database (GPRD), a large clinical database in the United Kingdom (UK).

## Methods

### Study population

The GPRD has been described in detail elsewhere[11,12]. Briefly, starting in 1987, selected general practitioners in the UK agreed to record electronically their patients' clinical information and provide these anonymized data for research purposes. Recorded information includes demographic variables, symptoms, lifestyles, such as smoking, clinical variables, such as body mass index, medical diagnoses, drug prescriptions, referrals to and diagnosis from specialists, and hospital admissions. Medical records, including hospital discharge and referral letters, are available for review upon request. Since its inception, more than 5 million UK residents have been included in the database. The GPRD population is representative of the British population with regard to age, gender, and geographic distribution[12]. Additionally, different validation studies have shown that data contained in the GPRD is of enough quality and completeness to be used for epidemiologic analysis[13-16].

The GPRD is managed by the Medicines and Healthcare Products Regulatory Agency in the UK. The present study has been approved by the Institutional Review Board at the University of Minnesota.

### ALS ascertainment

Cases of ALS were identified from the population of patients older than age 20 enrolled in the GPRD for at least two years from January 1990 to July 2008. Any participant with a computer diagnosis of motor neuron disease, ALS, progressive muscular atrophy, progressive bulbar palsy, or primary lateral sclerosis was considered a case. In a review of medical charts and death certificates of 65 potential ALS cases identified following this approach, we were able to confirm 85% of them[17]. Similarly, incidence rates of computer-diagnosed ALS in the GPRD were comparable to those found in population-based registries in Scotland and Ireland[17].

### Study design

To estimate the association of smoking with ALS risk, we conducted a nested case-control study. Cases were individuals with ALS with at least two years of follow-up before the date of diagnosis. Up to 10 controls free of ALS were selected for each case matched by age (± 3 years), gender, practice, and year of enrolment in the GPRD. Controls had to be alive and free of ALS at the date of diagnosis of their corresponding case (index date).

The association of smoking with ALS survival was assessed using a cohort design. Follow-up started on the date of diagnosis and continued until the patient died, transferred out to a different practice, or the end of July 2008, whichever occurred earlier.

### Exposure assessment

Information on smoking status before the index date was obtained from the computer database using the earliest smoking information for each study participant. Individuals were classified as never, past, or current smokers, or as having missing information. Detailed information on amount of smoking was not available but, as a surrogate marker of smoking intensity, we collected information on smoking cessation advice or treatment, and whether participants were labeled as 'heavy smokers'. In a previous publication, smokers receiving advice or treatment had a higher risk of lung cancer than other smokers, supporting the validity of this classification[18]. If a smoker in our population received smoking cessation advice or treatment, or was labeled as 'heavy smoker', we considered him or her to be a heavy smoker, and a non-heavy smoker otherwise. In the sample of controls, heavy smokers had a higher mortality than non-heavy smokers, and the non-heavy smokers had a higher mortality than never smokers, supporting the validity of our smoking classification: in an analysis adjusted for age and sex, the mortality rate ratio (RR) was 2.19 (95% confidence interval [CI] 1.71-2.80) in heavy smokers and 1.17 (95% CI 1.05-1.30) in non-heavy smokers compared to never smokers. Additionally, we obtained information on body mass index from the database. Information on smoking was missing in 18% of the study sample (19% in men and 17% in women).

### Statistical analysis

In the case-control study, we estimated the RR of ALS for smoking status, with never smokers as the reference group, via conditional logistic regression models adjusted for matching factors. The main analysis included all cases and their respective controls. We performed an additional analysis including only individuals with at least 5 years of recorded medical information before their index date. For this analysis, we advanced the index date by 5 years and only used smoking information recorded before this earlier date.

In the cohort study we estimated the mortality RR for smoking status via a Cox proportional hazards model with days from diagnosis to end of follow-up, as the time variable. In addition to smoking status, we included in the model the following variables: age at diagnosis, gender, diagnostic classification (ALS/motor neuron disease vs. ALS variants), and calendar year. Information on site of ALS onset was not available. Calendar year was modeled fitting a restricted cubic spline function with 3 knots. We excluded 12 cases in which date of diagnosis was the same as date of death. An additional analysis was conducted excluding individuals diagnosed with ALS variants. Adjusted survival was estimated in four groups defined by gender and smoking status (ever, never) averaging survival curves for each individual in the sample, using a SAS macro developed by Zhang et al.[19]

In both the case-control and cohort analyses, we estimated separate RR in men and women, and assessed their heterogeneity using the likelihood ratio test. Finally, we repeated all analyses including only individuals younger than 75 (800 cases, 8114 controls), since ALS diagnosis in older individuals is less certain.

## Results

During the study period, we identified 1143 ALS cases with at least two years of follow-up before their diagnosis, and selected 11,371 matched controls. Characteristics of cases and controls are presented in table 1. The median age in both groups was 67.

**Table 1:** Characteristics of amyotrophic lateral sclerosis (ALS) cases and controls, General Practice Research Database, 1990-2008

	ALS cases	Controls
N	1143	11,371
Age at index date, years	67.4 (12.5)	67.1 (12.5)
Range	23-95	20-97
Gender [% women]	44.8	44.9
Smoking [%]		
Never	33.0	32.7
Missing	16.7	18.0
Ever	50.3	49.4
Former	23.9	23.7
Current	26.4	25.7
Non-heavy smokers	45.1	44.5
Heavy smokers	5.2	4.9
Body mass index, kg/m^2 ^[%] *		
<20	3.6	3.2
20-24.9	33.1	30.1
25-29.9	29.5	30.7
≥30	9.5	11.9
Missing	24.3	24.2
Time between first smoking assessment and index date, years	14.1 (9.4)	14.0 (9.2)
Diagnosis [%]		
ALS/MND	88.6	
PBP	8.8	
PMA	1.4	
PLS	1.2	
Follow-up after diagnosis, years	2.1 (2.7)	

Numbers refer to percentages or means (standard deviation).

* Corresponding to first recording of body mass index in the GPRD (average 8.3 years before index date)

ALS: amyotrophic lateral sclerosis; MND: motor neuron disease; PBP: progressive bulbar palsy; PLS: primary lateral sclerosis; PMA: progressive muscular atrophy

The association of smoking with ALS risk is presented in table 2. Compared with never smokers, the RR (95% CI) of ALS for ever smokers was 1.04 (0.80-1.34). The corresponding RR (95% CI) was 1.53 (1.04-2.23) in women and 0.75 (0.53-1.06) in men. This gender difference was present in young and older individuals. In those older than 50, the RR (95% CI) of ALS for ever smokers compared with never smokers was 0.74 (0.51-1.07) in men and 1.49 (1.00-2.22) in women. In those 50 and younger, the RR (95% CI) were 0.84 (0.33-2.19) in men and 2.14 (0.51-9.04) in women.

**Table 2:** Rate ratios (RR) and 95% confidence intervals (CI) of amyotrophic lateral sclerosis (ALS) by smoking status before the onset of ALS, General Practice Research Database (GPRD), 1990-2008

	ALS cases	Controls	RR (95% CI) *	RR (95% CI)**
Entire sample				
Never	377 (33.0)	3714 (32.7)	1.00 (ref.)	1.00 (ref.)
Ever	575 (50.3)	5615 (49.4)	1.04 (0.80-1.34)	1.09 (0.81-1.46)
Former	273 (23.9)	2696 (23.7)	1.01 (0.74-1.39)	1.15 (0.80-1.64)
Current	302 (26.4)	2919 (25.7)	1.06 (0.79-1.41)	1.04 (0.74-1.47)
Missing	191 (16.7)	2042 (18.0)	0.88 (0.68-1.14)	0.94 (0.69-1.26)

Men				
Never	207 (32.8)	1896 (30.3)	1.00 (ref.)	1.00 (ref.)
Ever	318 (50.4)	3180 (50.8)	0.75 (0.53-1.06)	0.75 (0.50-1.11)
Former	157 (24.9)	1584 (25.3)	0.73 (0.48-1.11)	0.70 (0.43-1.14)
Current	161 (25.5)	1596 (25.5)	0.76 (0.52-1.13)	0.79 (0.50-1.24)
Missing	106 (16.8)	1191 (19.0)	0.64 (0.44-0.92)	0.67 (0.44-1.02)

Women				
Never	170 (33.2)	1818 (35.6)	1.00 (ref.)	1.00 (ref.)
Ever	257 (50.2)	2435 (47.7)	1.53 (1.04-2.23)	1.68 (1.08-2.61)
Former	116 (22.7)	1112 (21.8)	1.49 (0.92-2.43)	2.06 (1.20-3.53)
Current	141 (27.5)	1323 (25.9)	1.55 (1.01-2.38)	1.41 (0.84-2.36)
Missing	85 (16.6)	851 (16.7)	1.25 (0.85-1.82)	1.33 (0.86-2.04)
P interaction			0.01	0.02

* Conditional logistic regression model adjusted for age, gender, practice, and time since enrolment in the GPRD.

** Results advancing five years index date (910 cases and 9058 controls). Conditional logistic regression model adjusted for age, gender, practice, and time since enrolment in the GPRD.

Results were similar when we included only cases and controls with at least five years of follow-up before their index date (table 2), when we adjusted for body mass index (data not shown), and when we excluded individuals older than 75 on the index date: the RR (95% CI) was 0.77 (0.52-1.15) in men and 1.50 (0.96-2.35) in women. The RRs of ALS for heavy smokers and non-heavy smokers were of similar magnitude compared with never smokers. The RR (95% CI) of ALS in heavy smokers compared with never smokers was 1.08 (0.74-1.56) in the entire cohort, 0.72 (0.44-1.19) in men and 1.77 (1.02-3.09) in women. The corresponding figures comparing non heavy smokers with never smokers were 1.03 (0.80-1.33), 0.75 (0.53-1.06) and 1.50 (1.02-2.21).

**Figure 1:**
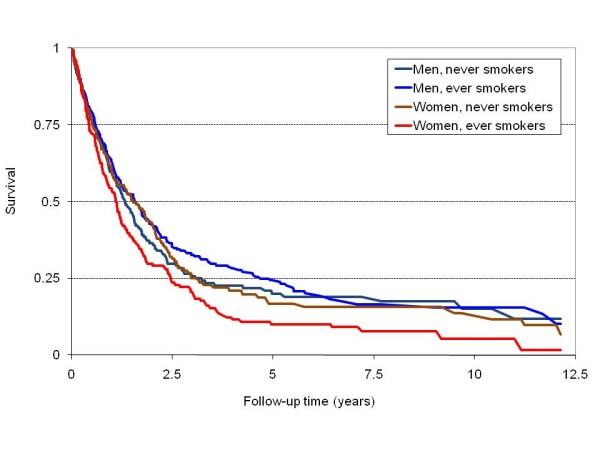
**Estimated probability of survival after diagnosis of amyotrophic lateral sclerosis (ALS) by gender and smoking status after standardizing for age, diagnosis (ALS vs. ALS-variants) and year of diagnosis, General Practice Research Database, 1990-2008**.

Of 1131 ALS cases with at least one day of follow-up after their diagnosis, 802 (70.9%) died, 108 (9.6%) transferred out, and 221 (19.5%) were alive at the time of last recording in the GPRD, after an average follow-up of 2.1 years. Median survival after diagnosis was 1.5 years. Old age and female gender were strongly associated with mortality. Each 5-year increment in age was associated with 20% higher mortality rate (RR 1.20, 95% CI, 1.16-1.24). Similarly, women had a 22% higher mortality than men (RR 1.22, 95% CI, 1.06-1.41). Compared with never smokers, the mortality RR (95% CI) was 1.45 (1.03-2.03) for heavy smokers and close to 1 for all other groups (table 3). Compared with never smokers, the mortality RR (95% CI) for ever smokers was 1.31 (1.04-1.65) in women and 0.90 (0.72-1.11) in men. The risk was particularly elevated in female heavy smokers (HR 1.94, 95% CI 1.24-3.06). As shown in figure 1, adjusted survival was lower in women who were ever smokers compared to men and women never smokers. Results were similar when we excluded individuals diagnosed with an ALS variant.

**Table 3:** Rate ratios (RR) and 95% confidence intervals (CI) of mortality in 1131 amyotrophic lateral sclerosis cases according to smoking status, General Practice Research Database, 1990-2008

Smoking status	N. deaths	Person-years	RR (95% CI) *
Entire sample			
Never	266	722.7	1.00 (ref.)
Ever	394	1103.3	1.06 (0.90-1.23)
Former	191	496.4	1.11 (0.92-1.34)
Current	203	606.9	1.01 (0.84-1.21)
Non-heavy smokers	352	1029.7	1.02 (0.87-1.20)
Heavy smokers	42	73.6	1.45 (1.03-2.03)
Missing	142	530.8	0.92 (0.74-1.14)

Men			
Never	141	400.9	1.00 (ref.)
Ever	213	733.4	0.90 (0.72-1.11)
Former	106	347.4	0.91 (0.71-1.17)
Current	107	386.0	0.88 (0.69-1.14)
Non-heavy smokers	195	695.8	0.88 (0.71-1.10)
Heavy smokers	18	37.6	1.08 (0.65-1.79)
Missing	76	282.8	0.91 (0.68-1.22)

Women			
Never	125	321.9	1.00 (ref.)
Ever	181	369.9	1.31 (1.04-1.65)
Former	85	149.0	1.46 (1.11-1.94)
Current	96	220.9	1.20 (0.92-1.57)
Non-heavy smokers	157	333.9	1.26 (0.99-1.59)
Heavy smokers	24	36.0	1.94 (1.24-3.06)
Missing	66	248.0	0.92 (0.67-1.26)

* Cox proportional hazards model adjusted for age, gender, diagnosis (ALS vs. ALS variant), and year of diagnosis.

## Discussion

In this large prospective study, smoking was a predictor or ALS in women, and of mortality in female ALS patients, but not in men.

Smoking has been associated with several neurodegenerative disorders. Prospective investigations have found a higher risk of dementia and a lower risk of Parkinson's disease in smokers compared to non-smokers[20,21]. The epidemiologic evidence regarding smoking and ALS is less consistent, with some studies showing a higher risk of ALS among smokers,[22-24] and others not finding any clear association[25]. Notably, in the Cancer Prevention Study II cohort, a prospective cohort including over 1 million people, results were similar to ours: ever smoking was associated with a higher risk of ALS mortality in women (HR 1.32, 95% CI 1.03-1.69) but with a lower risk in men (HR 0.77, 95% CI 0.59-1.00)[26]. Consistent with this observation, a recent systematic review and meta-analysis showed that the proportion of men and women in a particular study made a major contribution to between-study heterogeneity[7]. The estimated pooled RR of ALS in ever versus never smokers was 0.87 (95% CI 0.71-1.06) in men, and 1.58 (95% CI 1.21-2.08) in women[7]. Results from the GPRD are in agreement with the previous meta-analysis and reinforce its conclusions.

Smoking could increase the risk of ALS through different mechanisms. Strong evidence supports the role of smoking as a cause of oxidative damage,[2] and oxidative damage has been involved in the pathogenesis of ALS[27]. Also, more than 4 thousand different compounds have been identified in tobacco and cigarette smoke, including heavy metals, such as lead or cadmium, and formaldehyde[28]. Although the role of these in ALS pathogenesis has not been defined, some studies point to an increased risk of ALS and worse ALS survival associated with higher levels of lead exposure,[8,29] and to a potential higher incidence of ALS in individuals exposed to formaldehyde[30]. Systemic inflammation, another well-known effect of smoking, might also be involved[4]. Biological mechanisms supporting the difference in the association of smoking with ALS in the two genders are less clear, though prior observations suggest that women could be more sensitive to deleterious effects of cigarette smoking, probably through differences in the metabolism of tobacco compounds[31,32]. Additional evidence suggests that the association of smoking with different health outcomes, including lung disease or thyroid disorders, might differ by gender[32,33].

More intriguing is the sex difference in the association of smoking with survival. Only three previous studies have reported associations of smoking with ALS prognosis. Kamel et al studied the association of different exposures, including smoking, with survival in a group of 100 ALS cases in New England. In age and sex-adjusted analysis, ever smokers had a better survival than never smokers (HR 0.6, 95% CI 0.4-1.0, with time from diagnosis to death as the outcome, or HR 0.7, 95% CI 0.5-1.2, with time from onset of symptoms to death as outcome)[8]. In a report including 180 ALS patients from Washington State, smoking did not predict mortality: compared to never smokers, the HR of death in current smokers was 1.1 (95% 0.7-1.6) and 1.0 (95% CI 0.7-1.4) in past smokers[9]. Neither study assessed the association separately in men and women. Finally, smoking was not a significant predictor of survival in a group of 2069 ALS patients receiving riluzole in France, but the actual estimates of association were not reported[10].

Strengths of our study include the large sample size, providing enough statistical power for subgroup analysis, and the selection of cases and controls from a well-defined cohort, reducing the possibility of selection bias. Several limitations, however, might reduce the validity of our results. First, we have not confirmed all ALS cases identified in the GPRD, though a previous study suggested acceptable validity of the computer diagnosis[17]. Because validity of ALS diagnosis could be lower in older individuals,[34] we repeated analyses after excluding those older than 75, obtaining similar results. Second, smoking status was classified in broad categories (never, non-heavy smokers, heavy smokers, unknown). Thus, we were not able to determine precise dose-response relationships. Lastly, unmeasured confounding could explain our results if a risk factor for ALS was associated with smoking and distributed unevenly in men and women.

## Conclusions

We have found that smoking is associated with higher risk of ALS and worse survival in women but not in men. Future studies should focus on exploring the mechanisms that might explain the observed associations.

## Competing interests

The authors declare that they have no competing interests.

## Authors' contributions

AA performed statistical analysis, obtained funding and prepared the first draft of the manuscript. GL participated in study design and the interpretation of results. SSJ obtained the data and contributed to the interpretation of results. MAH participated in study design and interpretation of results, and obtained funding. All authors reviewed critically the manuscript for important intellectual content and approved the final manuscript.

## Pre-publication history

The pre-publication history for this paper can be accessed here:

http://www.biomedcentral.com/1471-2377/10/6/prepub
